# Medical Society of the County of Albany

**Published:** 1874-02

**Authors:** F. C. Curtis

**Affiliations:** Secretary


ART. II.—*Medical Society of the County of Albany.* Semi-Monthly Meeting, January 14th, 1874.

Reported by F. C. Curtis, M. D., Secretary.

The first Semi-Monthly Meeting of this Society for the winter was held as usual at the City Building, Dr. John Swinburne, President, in the chair. Thirty-two members were present. After the minutes of the last semi-monthly meeting had been read and approved, the attention of the Society was called to the death of Drs. J. F. Townsend, Peter Van Buren, and J. H. Lasher, former members, and committees were appointed to prepare suitable resolutions in regard to them.

Dr. E. R. Hun then reported a case of *Calculus in the Urethra*, as follows:

W. J., aged 53 year, came to me January 26th, 1873, complaining of a difficulty and pain in passing water. He told me that while in the British army twenty-four years ago, he contracted gonorrhœa for which he was treated by strong injections, which put an end to the discharge in a few days. He had no further trouble for the next ten years, when he suddenly found himself unable to pass water. He was relieved by catheterism, and for some time after had no further symptoms. About six years ago he observed that the stream of urine was becoming smaller, and that it took him a long time to empty the bladder, and some three or four years ago, after retaining his urine for some time and making forcible efforts to expel it he passed two or three small grity particles which occasioned very considerable pain. During the past three years he has never been able to pass water freely, and has several times suffered from retention, which has been relieved by warm baths and various internal remedies, advised by his friends. His urine now is expelled drop by drop, and his underclothing is constantly wet with that which passes involuntarily. He is considerably emaciated and so feeble as to be unable to do ordinary work; does not rest well at night, and has but little appetite. Upon attempting to introduce a silver catheter, I find it arrested about an inch from the meatus by a firm unyielding stricture. A No. 2 olive pointed bougie could be passed through this stricture, but encountered another about two inches farther back, through which it was passed with difficulty, although stiffened by the introduction of a wire stilet, a third stricture was met with in the membraneous portion of the urethra after passing which the instrument entered the bladder. Upon removing the bougie and and examining it, I was surprised to find it cut and scratch on its exterior, as if it had been drawn on some hard jagged body. I then felt along the urethra and found a calculus mass lying between the second and third strictures, which could be pushed up and down the urethra but was too bulky to pass the strictures. I endeavored to dilate the anterior strictures by graduated bougies, but it was so firm and unyielding that I made no headway.

A short time after I first saw the patient, the State Society met in Albany, and Dr. Otis of New York, exhibited his new urethrotome to me, which I thought would be just the instrument for such a case as the one aboved described.

I therefore asked him to meet the patient at my office, which he kindly consented to do, and we tried to introduced the instrument. The stricture however would not admit it and Dr. Otis then tried to enlarge the passage with a Maisonneuve's urethrotome. After considerable difficulty he succeeded in passing through the largest size blade of Maisonneuve's, and followed it with his own instrument, but not without using force, so dense and unyielding were the fibrous bands forming the two anterior strictures. The doctor then by means of the screw in the handle, opened the blades of his instrument so as to dilate the strictures preparatory to dividing it, but after a few turns of the screw the blades became clogged with the calculus material contained in the urethra, and he could neither open or shut it. He managed to withdraw it with great difficulty, and the pain occasioned by the process was so great that the patient refused to allow anything more to be done, and insisted upon going home.

I saw him the next morning, and found the penis much inflamed and swollen. He had passed only a few drops of urine, and the bladder was distended. I tried to induce him to let me perform external urethrotomy and remove the calculus and give exit to the urine, but he obstinately refused to permit anything to be done. He persisted in his refusal for the next few days, although in the mean time he suffered great pain, and the urine only dribbled away drop by drop. On the fifth day after Dr. Otis had seen him he consented to go to St. Peters' Hospital, where he was at once etherized, and Dr. Swinburne opened the urethra just in front of the posterior stricture. A calculus was removed measuring one inch in length by three-eighths of an inch in diameter, and with it three or four small angular masses of gravel. The stricture was then divided, and an elastic catheter passed into the bladder through which a large quantity of offensive urine passed off. The whole penis was sloughing from urinary infiltration, and the scrotum and several parts in the groin were incised and gave vent to purulent matter, having a strong urinous odor. The patient gradually sank and died forty-eight hours after the operation. No autopsy could be obtained.

From the size and shape of the calculus it must have been in the urethra for a long time, and it would be an interesting point to determine, whether it originally formed in the dilated portion of the urethra between the two strictures or forced from the bladder in the form of a small particle of gravel, and afterwards increased in size by the deposit of phosphatic matter from the urine, which was constantly retained in the pouch in which it was found.

I am inclined to the opinion, that the latter explanation is the true one, from the fact that other particles of gravel were found with it.

The experience of the case would lead me to at once perform external urethrotomy should a similar one present itself to me and not waste valuable time, and run the risk of urinary infiltration by attempting to dilate the strictures.

Dr. SWINBURNE remarked that the case was an unusual one. He had no doubt that it was strictly an urethral calculus, forming in the cul de sac between the strictures. He had never seen one just like it.

Dr. HENRY MARCH, spoke of five cases in which his father had removed calculi from the urethra. In one of these the patient had been troubled with symptoms of gravel for a year, when a small calculus became wedged in the urethra just in front of the scrotum. It was extracted by means of an instrument designed for the removal of a ball—a tripod spring in a canula. In the case of a child, the stone being located in the same situation was removed by external urethrotomy.

Another case occurred in the person of a shoemaker, aged 35. He was troubled for three years with the ordinary symptoms of renal calculus passing down the ureter. He has passed bloody urine, and had other symptoms of cystic irritation, and a calculus was detected in the bladder. He was put upon treatment preparatory to the operation of lithotomy, when the stone, half an inch in length and as large as a pipe stem was found to have passed into the urethra. It was removed by maniputution and the use of a large sound.

In all these cases the calculus originated in the bladder, passing thence into the urethra.

Dr. C. A. ROBERTSON read a paper entitled *Old Eyes made New*,* giving a case in which eye-sight was temporarily destroyed by the use of "eye cups" according to the advertisement of a charlatan.

Dr. Hailes enquired as to the location of the hemorrhage in the

* See Art. I.

case given. It could not be into the anterior chamber, as there it would be very apparent, and there could be no danger of mistaking it for a cataract.

Dr. Robertson said that he had not examined the eyes with the ophthalmoscope. It was probably due to a rupture of one of the small retinal or choroidal vessels.

Dr. J. P. Boyd, Jr., reported a case of Pneumonia as follows:

A few months ago a patient was admitted at St. Peters' Hospital, with the following history. Age 26; born in Ireland; occupation laborer. Stated that a few days before coming to the hospital he had a chill, followed by feverishness, and loss of appetite. Previous health had been good. On the evening of admission the temperature was 102°, pulse accelerated, respiration normal, otherwise nothing of interest. A laxative was prescribed, and the ordinary diaphoretic. The second night after admission he became delirious, and was with difficulty kept in his bed. He told the attendants at this time that "his bladder had burst" and that he could "not pass his water." Previous to this he had passed his water regularly, and without difficulty. A catheter was introduced by the house physician and a moderate quantity of highly colored urine drawn off. Nothing abnormal was discovered in the region of bladder, and urethra. Urine found to contain urates in abundance. Following morning temperature 104°, pulse 90. Face was now somewhat flushed; pupils normal. Continued delirious during the day. The delirium was of a good natured character, and patients gait on attempting to walk was unsteady.

On the evening of this day temperature, was 105°, pulse over 100; 4th day after admission temperature morning 103°, evening 105°; 5th day temperature morning, 103°, evening 103°.5; 6th day temperature, morning 103°, evening 105°; 7th day, morning 103°, evening 104°; 8th day, morning temperature 104°, evening 105°. Up to this date the urine had been drawn off regularly, the delirium had continued, although not so marked as at first. Respiration had been easy; patient had complained of no pain. On entering the ward at this date, I observed for the first time, that the breathing was hurried and that the lips were slightly cyanotic. He did not cough. On examining the chest, I found slight dullness over lower lobe of right lung, and not very marked dullness over left lung. Crepitant rales were heard on right side of chest. The chlorides were found to be diminished in quantity in the urine; 9th day, temperature morning 104°, evening 105°. Increasing dullness over lower lobes of both lungs; fine crepitant rales over both sides of chest. A herpetic eruption on the lips; increased cyanosis; cough for the first time; 10th day, temperature morning 105°, evening 105°.5; complete dullness over lower two-thirds of both lungs, bronchial breathing; broncophony, increased vocal fremitus; 12th day, temperature morning, 106°, evening 107°, this was the highest point realized in the disease. The characteristic rusty sputa of pneumonia was present. From this date the temperature fell, and slowly returned to the standard of health. The bronchial breathing, and broncophony gave place to the rales redux; the dullness gradually disappeared, and the lungs once more were in sound condition. Patient was in the hospital six weeks; confined to bed four weeks. Discharged cured. The treatment consisted in the use of the oil silk jacket, quinine, carb. of ammonia and nourishing diet. The points of interest in connection with the case are; 1st, the length of time existing between the chill and first symptom of trouble in the respiratory apparatus; 2d, the slowness of the pulse when compared with the high temperature of the disease, and to which the German authors call attention; 3d, absence of all pain, and cough until after disease had been fully established; 4th, the well marked stage of delirium.

Dr. Swinburne enquired concerning the experience of members as to means of reducing high temperature. He had himself found good result from filling the air of the room with steam.

Dr. Boyd said that he had seen in Germany, ice applied to the chest in Pneumonia, without unfavorable results.

Dr. Boyd also presented a pathological specimen of Fallopian Salpingitis. The specimen had been taken from a woman aged 25; married; never had borne children. She died in a fit of intoxication while suffering from Brights' disease. The liver was large and fatty; osseous deposits on the mitral and aortic valves. Membranes of the brain thickened and opaque. Kidneys both enlarged and fatty. Mucous membrane of the stomach dark red and congested, with evidence of chronic inflammation.

The uterus was of normal size; the mucous membrane of cervix and fundus in a state of chronic inflammation and covered with a thick tenacious substance which is found to be made up of pus corpuscles, fat and debris of epithelium. Very slight and beginning cell proliferation in tissues adjacent to the mucous membrane. The fallopian tubes admit a very fine probe for a distance of about three-fourths of an inch, beyond which point they are impervious. Under the microscope they present nothing special; the arborescent arrangement of folds of mucous membrane remains, but the epithelium is destroyed and pus corpuscles are numerous. About an inch from their uterine mouth both tubes are widely dilated and terminate in blind sacs near the ovaries. The fimbriated extremities cannot be recognized. The abdominal extremities of both tubes are bound by firm adhesions to ovaries. The mucous membrane of the dilated tubes is replaced by a smooth, shining membrane consisting mainly of cellular tissue and covered by some cells of flat form; the remaining coats consist mainly of cellular tissue. The contents of each tube weighs two ounces, and is a greenish, gelatinous substance which on examination is found to contain pus, fat and granular bodies. The ovaries are of normal size, and on section, show numerous small cysts. Both are firmly bound to the tubes.

The subject of providing an entertainment for the State Society, at its next annual meet was brought up by Dr. W. H. Baily. The question was discussed at some length, and the Society finally adjourned with action being taken upon it.

---

January 28th, 1874.

The society met at the usual hour and place. Dr. Swinburne, President, in the chair.

About thirty members were present.

The names of the following gentlemen were proposed for membership, and referred to the *comitia minora;* Drs. L. T. Morrill, G. L. Van Allen, W. W. McGregor, H. C. Everts and A. T. Van Vranken.

Dr. L. R. Boyce presented a specimen of an *anencephalic monster*, with a sketch of the case. He said: In the latter part of November last, I was called to attend in confinment Mrs. R., aged thirty-five. She is the mother of five children, born at full term and well formed, only one of which is now living. Two died at two or three years of age, one nine days after birth, and one was still-born. She also had a miss-carriage at the third month, a year and a half ago. The present confinment came in at the seventh month of pregnancy. She has always enjoyed excellent health until within the past two years, when her strength declined from exposure and hard work, which she was obliged to undergo on account of her husband being thrown out of employment. Having never been accustomed to such outside work, and being, as she thought, very hardy, she exposed herself so much that she caught severe colds, and although never stricken down with any particular disease, she has been, as she expresses it, "delicate" ever since. She is nervous, bowels irregular, appetite capricious, and though looking strong and fleshy she can endure but little work or exercise. Her two miss-carriages have occurred since this breaking down of her health and strength.

On reaching the bedside, I found that she had felt no life for three days; that the membranes had given way some hours before and the pains were light and regular every five minutes. The os was well dilated and the head presenting. You can imagine from the appearance of the head of the fœtus, the difficulty in deciding at that moment as to the presentation. Labor proceeded regularly and ended in about two and a half hours after I first saw her. The placenta passed off readily and she made a good recovery, no sequellae following.

I have not looked up the subject of monstrosities, but I suppose the condition of this fœtus, due to a lack of development from defective nourishment on account of ill-health of the mother.

The head of the fœtus is set squarely on the shoulders, the neck being almost wanting. It slopes back directly from the eye-brows and the brain is altogether wanting. Spina bifida is also present.

Dr. Moore remarked that he had a similar case several years ago. The parents were both healthy, and well developed children had been produced by them both before and since the birth of the monster.

Dr. Beckett said that he had also met with one of the same nature. He noted the fact that the liquor amnis was excessive in quantity, fully a pailful passing away. The same has been observed by others, and the cause of the undeveloped condition of the fœtus has been looked for in an abnormal condition of the amniotic membrane.

Dr. Levi Moore presented the following case of rupture of the heart, with the pathological specimen:

Mr. R., has suffered from a stricture of the urethra for more than thirty years, and his urine had been voided with pain and difficulty. The cause of this stricture could not be ascertained, nor did I learn that any efforts were ever made to effect a cure by the usual methods of dilatation. About seventeen years ago, owing to the presence of a foreign substance or to inflammatory action, sloughing of the penis took place, a false passage was found through which the urine flowed, and considerable destruction of the neighboring tissues by gangrene ensued, placing his life in imminent peril. He made a slow and tedious recovery. The fistulous opening healed slowly without affording relief to the stricture. He also suffered for many years from hemorrhoids. About two weeks before I was called to attend him, he complained of severe pain through the left side and shoulder, which was aggravated by exercise. He also had occasional attacks of dyspnœa.

I was summoned to attend him Nov. 2d, 1873. I found him suffering from great prostration, with a weak irregular pulse, severe cardiac pain extending to the left shoulder, and difficult respiration. I was not able to detect any organic lesion of the heart. By the aid of anodynes, a nutritious diet and absolute rest, the condition of the patient was much improved, and the depression of mind which had marked the case when I was called, gave way to a feeling of cheerfulness and hope of an early recovery. The apparent improvement in the condition of the patient continued until Nov. 12th, when after taking a walk of two or three blocks and returning, he complained of great fatigue, a return of pain in the region of the heart, and expressed himself as feeling very badly. He passed a restless night, and the next morning while seated in a chair suddenly expired.

The autopsy was made by Dr. Van Derveer, in the presence of several of the physicians of the city. The left ventricle of the heart was found ruptured near the base, and the pericardium was distended with a large clot and about six ounces of serum. The heart was enlarged, and its muscular tissue flabby and pale in appearance. There was considerable effusion into the pleural and abdominal cavities.

An examination of some of the facts connected with rupture of the heart may not be without interest. It occurs more frequently in males than females, in the proportion of about five to two. In most of the reported cases it occurred at an advanced age, often between fifty and sixty, more frequently after the sixtieth year.

When this lesion takes place we may expect to find in most cases that some diseased condition of the heart previously existed. Fatty degeneration with softening of the muscular tissues of the organ is perhaps the most frequent condition. Calcification of the ruptured tissues is often found, or, this wanting, we may look for some one of the many changes produced by disease in the structure of this organ.

It has been observed, that in most cases of rupture of the heart, as in the case I have presented, the rupture takes place in the left ventricle. Next in order it has been found that the right ventricle is involved; much less frequently the auricles.

In most cases of rupture of the heart, the lesion occurs suddenly, and is attended with almost instantaneous death; in other cases however, the rupture appears to take place more slowly, the patient has pain in the precordial region extending to the shoulder, dyspnœa, giddiness and faintness with small and perhaps irregular pulse. These symptoms, more or less urgent and always increased by exercise, may continue through several weeks, while the muscular fibres are gradually yielding to the strain to which they are subjected, until the rupture becomes complete, when death at once ensues.

To this latter class I believe, belongs the case I have presented to you this evening. Well marked cardiac symptoms were observed nearly four weeks before death, due no doubt to the rupture of a layer of muscular fibres. The graver symptoms which existed when I was called to see him, resulted I believe from an increase of this lesion, by the rupture of additional fibres, and two weeks later the rupture became complete, when death instantly ensued.

It may be fairly a question if, in the case of Mr. R., the stricture of the urethra, with its attending straining in his efforts to empty the bladder, may not have contributed something towards producing the lesion of the heart which finally took place. Indeed as there was no marked strictural change in the vicus itself, is it not probable that such was the case?

Dr. Beckett reported having met with a case of rupture of the heart, in which no extraordinary exertion had been made, the patient having died while in bed at night. There had been no immediate symptoms that were at all urgent preceeding it. An opening hardly the size of a quill was found in the right auricle. The valves were very extensively calcified. The subject had always been a healthy man.

Dr. Lansing reported a similar case which had occurred recently. The patient, an elderly gentleman, having been previously in his usual good health, was taken at four o'clock in the morning with precordial pain and dyspnœa. He arose from bed and went to a window for air. The pain ran down both arms to the ends of the fingers. Ether, carbonate of ammonia and hypodermic injections of morphia were administered, with the effect of relieving him in a measure. Next day while sitting at stool he died suddenly.

There was found, *post mortem*, a firm coagulum investing the heart and distending the pericardium. There was no serum. An inch from the apex of the right ventricle there was a large rent. about the point of rupture an apoplectic effusion had taken place. The coronary arteries were atheromatous, and one was opened at the point where it crossed the rent in the heart. The heart was fatty and large, weighing upwards of thirty ounces. A point of interest in the case is when the rupture took place. It would seem that it began when the first symptoms appeared, and was not complete until next day, when the patient died while straining at stool.

Why did rupture take place in this heart? It is usually associated with exertion more or less violent, but there was no such cause here at first. The fatty condition usually existing was present. Is it not probable that it took place from muscular contraction of the heart, which is strongest at the apex.

What is the cause of death in rupture of the heart? Is it pressure on the organ, or shock, or loss of blood from the circulation? The two latter would seem to be the main elements, for the heart is often subjected to great pressure by pericardial effusion without fatal issue. Attention was called to the fact that there was no serum, but simply a firm clot in the pericardium. In all the cases he had seen, the same condition was found.

Dr. Mosher spoke in regard to the cause of death in rupture of the heart. He mentioned a case of rupture of the aorta within the pericardium, in which the blood was probably escaping into the pericardium for about ten hours. The patient having eaten freely was taken with unpleasant feelings and pain, which were ascribed to indigestion. A cathartic was taken without producing relief. When seen a few hours before death, he was laboring under extreme dyspnœa, nervous excitement, exhaustion and syncope. No distinct action of the heart could be made out. There was found, after death, a quart of solid and fluid blood in the pericardium, completely distending it, and practically obliterating all the cavities of the heart. Doubtless all the symptoms were produced by the occurrence of the rupture, and the consequent escape of blood. The cripling of the heart, and the loss of so large a quantity of blood from the circulation, were, either of them, sufficient causes of death, but probably the former was the main element.

Dr. J. S. Bailey reported a case of rupture of the heart, occurring in the person of an elderly female. She had been affected for years with dyspnœa on extertion. Twenty-eight hours before death, she was taken suddenly with syncope, and complained of a tearing sensation about the heart. Her countenance was blanched, pupils dilated, skin was cold, pulse scarcely perceptible and no heart sounds distinguishable. There was found a rupture of the ascending aorta half an inch above its origin, the internal coat being first perforated and dissected up a short distance, before tearing through of the external coats took place. There was about a pint of solid and fluid blood in the pericardium.

Dr. VAN DERVEER reported two cases, presenting the specimens; one of rupture of the aorta, similar, to Dr. Bailey's, and one of rupture of the heart.

RUPTURE OF THE HEART.—J. R., aged 63. Had usually enjoyed good health, with the exception of obstinate constipation, and this was relieved by an injection each morning.

On Dec. 25, 1871, having passed a comfortable night, he got up at his usual hour; made no complaint of feeling ill until after he had taken an enema at a little past 7 A. M. At this time he experienced great pain in the abdomen, more especially in the umbilical region, and becoming more and more severe, though under medical treatment, yet little relief was afforded. At 9:15 A. M. he suddenly referred his pain to the region of the heart, and in a severe paroxysm died.

Post-mortem was held at 3 P. M. All the organs of the abdomen presented a healthy appearance. The ascending transverse and a portion of the descending column was very much contracted. On laying open the intestine it did not appear that the calibre or cavity was larger than a pipe stem. On opening the thorax the lungs presented a healthy appearance. The pericardium was observed to be greatly distended. On opening, it was found filled with partly coagulated blood; in all, estimated to be eight ounces. There was a rupture of the anterior walls of the left ventricle near its base, about half an inch in length. The heart appeared pale, yet firm in texture; was of normal size, and presented no disease of its valves.

RUPTURE OF THE AORTA.—L. U., aged 49, a musician, had for a long time complained of feeling badly in the region of the heart. On the evening of March 1st, 1870, having passed an unusually restless day, he visited the Opera House, where he was engaged to play; but soon after commencing, was observed to turn suddenly pale, to cease playing, and in a moment to fall forward upon the floor. He expired immediately, before any medical assistance was rendered.

At the the time no *post-mortem* was allowed by the family; but three weeks later, the body having been well frozen and placed in the vault, they consented.

On opening the thorax and pericardium, the latter was found distended with blood. On examination, the inner coat of the arch and descending portion of the aorta was found studded with atheromatous deposits. About three inches from the heart the inner coat of the aorta had given away. The blood had dissected its way down for half an inch, and then had burst through the middle coat, and having worked its way down between the middle and external coat had ruptured through the latter just within the pericardium. The aorta appeared large, but there was no distinct aneurismal sac. The heart was hypertrophied, and the semilunar valves of the aorta were approaching ossification. There was no other disease of the remaining valves. No other organs were examined.

The President enquired if death had occurred, in the experience of any of the members, except at an advanced age, or upwards of fifty. He had not seen it. This would be an important consideration in forming an opinion in certain cases.

Several gentlemen expressed opinion coinciding with this.

Dr. Craig thought it evident that rupture of the heart is more common than formerly. A number of specimens were presented at this meeting, most of them having occurred recently. He raised the question as to the cause for this.

Dr. Van Derveer thought that this was more apparent than real, due probably to more post mortem examinations being made now than formerly.

Dr. J. H. Blatner read a paper entitled "Cases in Obstetric practice."

He first presented an analysis of one hundred cases as follows:

| | |
|---|---|
| Left occipito—anterior, | 49 |
| Right " " | 30 |
| Placenta praevia centralis, | 1 |
| Placenta praevia lateralis, complic with shoulder present. | 1 |
| Lateral plane with prolapse of hand and arm, | 1 |
| Prolapsed uterus, | 1 |

| | |
|---|---|
| Placenta accerta, | 4 |
| L. O. A. complicated by tumor at junction of coccyx and sacrum, | 1 |
| Twins, complic. with lacerat. of cord, | 1 |
| Cord about neck of child, | 4 |
| Face presentation, | 2 |
| Eclampsia complicating labor, | 1 |
| Constrict. band from sup. to inf. com. complic. labor | 1 |
| Hand and arm prolapsed aside of shoulder, | 2 |
| Abortion in consequence of typhoid fever, | 1 |

The following are a few cases of interest from the number:

CASE I.—*Placenta praevia centralis.* Mrs. P., aged 22, robust constitution, primipara. Had hemorrhages during the 3d, 4th, 5th and 7th months of pregnancy. The hemorrhage being persistent during the latter month the diagnosis of placenta praevia was made. At this time labor pains also set in, and the hemorrhage continued. Making little progress after twelve hours of labor, and having employed the tampon and a rubber bag filled with water to check the hemorrhage, but with little success, we concluded to dilate the os and cervix with Barnes' dilator. The dilators were used during the day, when the os being sufficiently open to admit the passage of two or three fingers, rapid dilatation was effected by means of the fingers and hand. The insertion of the placenta being directly over the os it was almost entirely removed, and podalic version performed, very little hemorrhage following the operation. After the delivery of the fœtus a small portion of placenta was found to be adherent to the inferior margin of the anterior surface of the uterus. The hemorrhage following the operation was not such as we should naturally expect with an adherent placenta. I attributed the recovery of this case to the method of treatment followed, viz: the rapid dilatation of the os and cervix, and the quick termination of labor, the *accouchment force* of the French writers. A few days after confinment parametritis developed itself, followed by pelvic cellulitis and the formation of a pelvic abscess. The abscess finally opened through the posterior wall of the vagina. The remaining history of the case is that of a slow recovery. The patient is now in good health, and has since mis-carried again at the fifth month of pregnancy. The treatment of the complications attending the lying-in state, was by carbolic acid injections, and a general tonic treatment and regimen.*

CASE II.—*Shoulder presentation, complicated with placenta praevia lateralis.* Mrs. G., aged 35, robust constitution, multipara. Had at her first labor twins, which were still-born, and at her second a macerated fœtus. During this, her last confinement, pains came on early and no diagnosis of the presentation could be made during the first twenty-four hours of labor. After a siege of nearly forty-eight hours, the following condition of things was found. The shoulder was presenting at the os, and to one side the margin of the placenta could be easily distinguished. Auscultation was repeatedly employed, but it gave no clue as to whether the child was living or dead. The pelvis being somewhat contracted and the patient fast failing in strength, podalic version was determined upon and performed while the patient was under the influence of chloroform. After considerable trouble in turning, owing to the escape of the amniotic liquor, an asphyxiated child was delivered, which could not, however, be resusitated. The placenta was found to be attached low down and partially at the inferior margin of the uterus and os internum. Considerable hemorrhage followed the removal of the placenta which was partially adherent, but it was controlled by means of pressure, ergot, cold applications, etc. The patient rallied well while in child bed, and is at present in good health. The only objection to version in this case might have been the possibility of detaching the placenta during the operation, and thus engendering a dangerous hemorrhage; but the position of the child and the late hour at which a diagnosis was made in this case. admitted of no other course of proceedure. Should a similar case occur again in my practice, I would, providing I could make a diagnosis sufficiently early, employ rapid dilatation, and effect as speedy a delivery as possible, in order to save the life of the child, the cause of whose death in the case cited, was

* Molesworth's uterine dilator was shown—an instrument for the rapid dilatation of the os and cervix. It consists of a piston syringe to which is attached a gloove finger shaped rubber bag, which being injected with water, admits of considerable expansion. It was said to have acted satisfactorily.

undoubtedly owing to the long continued pressure upon the placenta and cord

CASE III.—*Left lateral plane—presentation with prolapse of the arm and hand.* Mrs. D., aged 40, pale and anæmic, multipara; has had six previous confinements most of which were breech presentations. At my first examination which was twelve hours after uterine contractions had begun, and the waters broken, I found a hand and arm filling up the vagina, and upon introducing my finger into the os, plainly felt the thorax of the child. By external manipulation I found the head in the left side of the mother. Examining the position of the hand which lay with its palm toward the anterior surface of the vagina, I diagnosticated a lateral plane presentation, with the abdomen of the child toward the anterior surface of the vagina. The pulsation of the fœtal heart was heard on the left side below the umbilicus, and at the rate of 132 per minute. Podalic version was the only plan of treatment indicated, and it was accordingly performed with much difficulty and only after repeated attempts. The extremities and thorax were easily brought down as far as the head, which would not yield Smellie's method of hooking the finger in the mouth of the child was first attempted, but proving unsuccessful I employed the method generally known under the name of the "Prague manipulation." This consists in bringing the body well down, placing the first and third fingers aside of the nape of the neck, bringing the occiput under the symphysis by drawing the body of the child well towards the nates of the mother, then raising the body towards the abdomen of the mother by means of which the forehead and face are carried over the perineum. The only objection to this treatment is the impossibility of supporting the perineum, as both hands of the operator are in use. The danger of tearing the head from the trunk, as stated by some authors, is hardly I think to be dreaded. In case of a macerated fœtus it might possibly occur. By this manipulation I succeeded in delivering the fœtus which proved to be, as I had suspected from the fœtal pulsation, a male child, with an unusually developed, although not hydocephalic head. The patient progressed toward convalescence without any interruption. The child which was born in an asphyxiated condition was resuscitated by introducing a small catheter into the larynx and thus inducing artificial respiration.

In regard to the diagnosis of sex in utero by means of auscultation of the heart, twenty cases have been tested, and a correct diagnosis made in fourteen. Of five female children, the pulse ranged from 135 to 180; and of nine males from 110 to 130.

Case IV.—*Labor impeded by a bony tumor at the junction of the sacrum and coccyx.* Mrs. R., aged 40; weak constitution; has had two previous confinements, the first of which was tedious but terminated naturally; at the second she was attended by a homœopathic physician in Brooklyn, who applied the forceps after she had been in labor for over forty-eight hours. Patient states that when the child was delivered with the forceps she felt something snap or break. At her third confinement in which I attended her the pains came on well, and the os dilated rapidly. Everything progressed normally until the head had fairly entered the excavation of the pelvis, when it seemed to be impacted. Upon making a more careful examination of the bony structure of the pelvis, I found a hard resistent body about the size of a walnut, with one end flattened, just at the junction of the sacrum and coccyx, which evidently was the cause of the non-advancement of the head. Upon questioning the patient more closely I discovered that she had sustained a fall upon her back and buttocks when seventeen years of age, and was lame in consequence for some time after. The tumor was apparently caused by the union of a fracture of the lower end of the sacrum with resulting exuberent callus; and causing anchylosis of the sacro-coccygeal joint. After consultation with Dr. Van Derveer, we decided to apply the forceps. The head being small was easily delivered without any unpleasant complication. With the exception of a very lame back the patient convalesced nicely. The head of the child bore no impression whatever, and it is to this day living and in good health.

Case V.—*Fragile Cord—Ligature applied three times.* Mrs. M., a healthy multipara. The case was a perfectly normal one, and nothing untoward happened until I proceeded to ligate the cord, which as is my custom, was tied about three inches from the navel. The first ligature which was not tightly drawn cut through the coats of the umbilical vessels, and copious hemorrhage ensued. I immediately applied a second ligature, but with the same result. The ligatures which I had used were composed of five or six strands of cotton threads. and a serviceable one as I have found in most cases. Compressing the bleeding vessels with my fingers, I sent for some broad tape, and finally succeeded in ligating the cord by means of it, there only remaining about one inch of the cord attached to the umbilicus. Was this a case of fatty degeneration of the umbilical arteries and veins? It certainly demonstrates the necessity of not cutting the cord too near the navel.

CASE VI.—*Twins at seven months, complicated with laceration of the Cord.* Mrs. F., aged 28, multipara; called in great haste by the messenger, stating that "the woman was bleeding to death." When I arrived, I found the patient very feeble, and almost as pale as a cadaver. The bed was drenched with blood. Upon inspection I discovered a seven months fœtus already born, and immediately tied the cord. Examining per vaginum, I felt the breech of a second fœtus, which was not advancing, as there was no uterine contractions, and the uterus was in a state of atony. There being no time to lose, I extracted the second fœtus, and in doing so must have lacerated the cord, for there was very profuse hemorrhage, which I afterwards found came from the laceration of the cord. The placenta almost immediately followed. The hemorrhage ceased upon exerting pressure above and below the laceration, until I had an opportunity to apply the ligatures. The placenta was divided in two parts by a membranous bridge. The patient needing all my attention, I could make no attempt to resuscitate the children. She made a very slow recovery, and is yet suffering from all the effects of anæmia.

Dr. MOSHER said that he had been much interested in the paper; such analyses of cases being always of benefit. In regard to placenta praevia, he thought the main thing is to attend to the case without delay; the great danger to both mother and child being from loss of blood. He had found no difficulty when the os was dilated to the size of a silver half dollar in dilating it rapidly with the fingers and hand; when the child should be delivered as soon as possible. This is the only sure way to stop the hemorrhage.

Dr. BABCOCK said that his experience in regard to rapid dilatation coincided with that of Dr. Mosher. As to cutting the cord with the ligature, he had used bobbin and strands of cotton thread, and found that both of them sometimes did this. A substance softer than these should be used.

He also presented a bit of rough bone about two inches long, apparently from beef, which he had removed from the rectum of a patient. The man had presented himself at the alms-house, having been traveling some distance afoot. The foreign substance had set up a great deal of irritation, all the tissues about being swollen and inflamed. No clear history of the case was obtained.

On motion, the Society adjourned.

